# Quantitative analysis of ^99m^Tc-pertechnetate thyroid uptake with a large-field CZT gamma camera: feasibility and comparison between SPECT/CT and planar acquisitions

**DOI:** 10.1186/s40658-023-00566-3

**Published:** 2023-07-31

**Authors:** Benjamin Serrano, Régis Amblard, Tiffany Beaumont, Florent Hugonnet, Matthieu Dietz, Frédéric Berthier, Nicolas Garnier, Rémy Villeneuve, Valérie Nataf, François Mocquot, Christopher Montemagno, Marc Faraggi, Benoît Paulmier

**Affiliations:** 1 Medical Physics Department, Centre Hospitalier Princesse Grace, Monaco, Monaco; 2 Laboratoire d’évaluation de la dose interne, Institut de Radioprotection et de Sûreté Nucléaire, Fontenay-aux-Roses, France; 3 Nuclear Medicine Department, Centre Hospitalier Princesse Grace, Monaco, Monaco; 4 Nuclear Medicine Department, Hospices Civils de Lyon, Lyon, France; 5 Department of Biostatistics, Centre Hospitalier Princesse Grace, Monaco, Monaco; 6 Department of Medical Biology, Centre Scientifique de Monaco, Monaco, Monaco

**Keywords:** Thyroid, Absolute activity, Uptake, Quantification, CZT, Planar, SPECT/CT

## Abstract

**Purpose:**

The main objective of this study was to evaluate the ability of a large field Cadmium Zinc Telluride (CZT) camera to estimate thyroid uptake (TU) on single photon emission computed tomography (SPECT) images with and without attenuation correction (Tomo-AC and Tomo-NoAC) compared with Planar acquisition in a series of 23 consecutive patients. The secondary objective was to determine radiation doses for the tracer administration and for the additional Computed Tomography (CT) scan.

**Methods:**

Cross-calibration factors were determined using a thyroid phantom, for Planar, Tomo-AC and Tomo-NoAC images. Then Planar and SPECT/CT acquisitions centered on the thyroid were performed on 5 anthropomorphic phantoms with activity ranging from 0.4 to 10 MBq, and 23 patients after administration of 79.2 ± 3.7 MBq of [^99m^Tc]-pertechnetate. We estimated the absolute thyroid activity (AThA) for the anthropomorphic phantoms and the TU for the patients. Radiation dose was also determined using International Commission on Radiological Protection (ICRP) reports and VirtualDose^TM^CT software.

**Results:**

Cross-calibration factors were 66.2 ± 4.9, 60.7 ± 0.7 and 26.5 ± 0.3 counts/(MBq s), respectively, for Planar, Tomo-AC and Tomo-NoAC images. Theoretical and estimated AThA for Planar, Tomo-AC and Tomo-NoAC images were statistically highly correlated (*r* < 0.99; *P* < 10^–4^) and the average of the relative percentage difference between theoretical and estimated AThA were (8.6 ± 17.8), (− 1.3 ± 5.2) and (12.8 ± 5.7) %, respectively. Comparisons between TU based on different pairs of images (Planar vs Tomo-AC, Planar vs Tomo-NoAC and Tomo-AC vs Tomo-NoAC) showed statistically significant correlation (*r* = 0.972, 0.961 and 0.935, respectively; *P* < 10^–3^). Effective and thyroid absorbed doses were, respectively (0.34_CT_ + 0.95_NM_) mSv, and (3.88_CT_ + 1.74_NM_) mGy.

**Conclusion:**

AThA estimation using Planar and SPECT/CT acquisitions on a new generation of CZT large-field cameras is feasible. In addition, TU on SPECT/CT was as accurate as conventional planar acquisition, but the CT induced additional thyroid exposure.

*Trial registration* Name of the registry: Thyroid Uptake Quantification on a New Generation of Gamma Camera (QUANTHYC). Trial number: NCT05049551. Registered September 20, 2021—Retrospectively registered, https://clinicaltrials.gov/ct2/show/record/NCT05049551?cntry=MC&draw=2&rank=4.

## Background/introduction

For more than 50 years, sodium ^99m^Tc-pertechnetate (^99m^TcO_4_) has been used to assess thyroid function and thyroid uptake by planar thyroid scintigraphy [1–6]. ^99m^TcO_4_ has been used worldwide for thyroid gland examination because it has a short half-life (6 h) compared to ^131^I-Iodide (8 days) and because it does not produce beta radiation, providing a low overall radiation dose to the gland [7]. ^123^I-Iodide has similar advantages to ^99m^TcO_4_ in planar scintigraphy, namely a short half-life (13 h) and a gamma-ray emission of 159 keV compared to the 140 keV of ^99m^Tc, both of which are suitable for an Anger camera. However, cost and availability factors have made ^99m^TcO_4_ preferred over ^123^I-Iodide.

Positron Emission Tomography (PET) with ^124^I has recently been presented as a valuable clinical tool for the exploration of patients with thyroid disease [8, 9]. Darr et al. [10] performed a blinded pilot comparison between PET with ^124^I and planar ^99m^TcO_4_ scintigraphy or its cross-sectional enhancement single photon emission computed tomography (SPECT) for thyroid characterization. The conclusion of the study provides superior imaging of PET with ^124^I due to higher spatial resolution. However, the lack of commercial availability of the tracer, the potential cost of ^124^I and the need to use a PET camera are significant limitations compared to ^99m^TcO_4_.

Tomographic acquisitions (SPECT and SPECT/CT) are powerful diagnostic tools that improve the diagnostic quality of conventional planar scintigraphy which is currently the gold standard. Absolute quantitative analysis has proven to be achievable in SPECT/CT [11–14], thanks to technical improvements such as iterative reconstruction, scatter correction, computed tomography (CT) attenuation correction and resolution recovery [15, 16].

A new generation of gamma cameras with Cadmium Zinc Telluride (CZT) dual detectors with a large field of view have recently emerged. These cameras offer better spatial and energy resolutions than NaI scintillator detector [17]. All-purpose CZT cameras have been introduced to the market, but they are not yet widely used to study thyroid disease. Our aim was to investigate thyroid quantification (absolute thyroid activity and thyroid uptake) in anthropomorphic phantoms and in patients with planar and SPECT imaging on a latest generation of CZT gamma camera, with and without attenuation correction using a low dose CT scanner. We also aimed to investigate the respective fraction of thyroid absorbed dose caused by the CT scan and the isotope administration.

## Materials and methods

### Data acquisitions

All acquisitions were performed using a dual-head gamma camera Discovery NM/CT 870 CZT (GE Healthcare, Milwaukee, WI, USA). The camera was equipped with a WEHR45 (Wide Energy High Resolution 45 mm length) low energy collimator adjusted to the CZT detector element with pixel size of 2.46 mm. The collimator description is of a square hole type with a length of 45 mm, side of 2.26 mm and septal thickness of 0.2 mm. Clinical acquisition protocols for planar and SPECT/CT imaging are detailed below. The acquisition analysis was carried out on XELERIS 4.1 processing software (GE Healthcare) workstation with Q-Volumetrix MI post-treatment application. To date, there is no pinhole on this equipment.

*Planar* images over antero-posterior incidence of cervical region were acquired with the following camera settings: 256 × 256 matrix size, zoom factor of 3, acquisition time of 900 s and a photon energy window of 140 keV ± 10%. The pixel size was 0.74 × 0.74 mm^2^. The distance between the anterior head of the camera and the patients or phantoms was fixed at 12 cm. An advanced settings algorithm, called Clarity Zoom (GE Healthcare) was applied. It is described as a post-processing imaging enhancing system that works by filtering and resampling images with contrast enhancement. The planar acquisition was considered as the reference image for the thyroid uptake estimation in clinical practice [1–6, 18, 19].

*Tomography* acquisitions were performed with a 128 × 128 matrix size, a corresponding voxel size of 4.92 × 4.92 × 4.92 mm^3^, 60 projections (30 per detector head), 20 s per projection and zoom factor 1. We used the option of acquiring during motion between steps inducing a total time acquisition of 700 s. An energy spectrum window of 140 keV ± 7.5% and a scatter window of 120 keV ± 5% were used. The autocontouring (body contour) option was selected to ensure a minimum distance between detectors and the phantoms or the patients. Data of SPECT images were reconstructed using an iterative Ordered Subsets Expectation Minimization (OSEM) reconstruction algorithm, 4 iterations and 6 subsets, which included resolution recovery reconstruction (RR) and scatter correction. A post-reconstruction filter was also applied: a Gaussian filter of 1.5 mm in *X*, *Y*, and *Z* directions. The attenuation correction was applied using the CT scanner, a 16-slice Optima CT540, for the Tomo-AC dataset. Another set of reconstructions was made with the same parameters but without attenuation correction (*Tomo-NoAC*).

CT images were acquired from the maxilla to the sternal manubrium using the following parameters: 100 kVp tube voltage, tube current modulated with Smart mA (GE Healthcare, Milwaukee, WI, USA), maximum 75 mA and noise index up to 30, rotating time 0.9 s, pitch 1.375, 512 × 512 matrix size and slice thickness of 1.25 mm. The voxel size was 0.98 × 0.98 × 1.25 mm^3^. An iterative reconstruction algorithm, Adaptive Statistical Iterative Reconstruction (ASIR), (GE Healthcare, Milwaukee, WI, USA), was used for the data reconstruction.

### Cross-calibration parameters of the CZT gamma camera

**Fig. 1:**
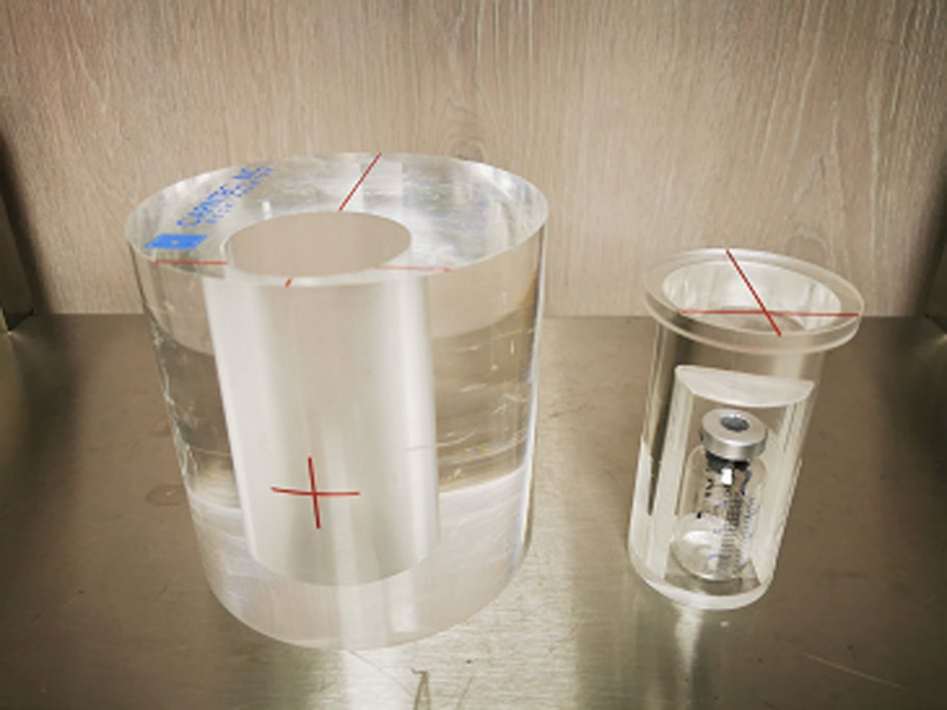
Neck phantom for thyroid cross-calibration factor determination for the planar, SPECT/CT and SPECT acquisitions

The cross-calibration factor was computed using the acquisition of known ^99m^Tc activity in a standardized phantom, a Plexiglass cylindrical ANSI/IAEA (American National Standards Institute/International Atomic Energy Agency) neck thyroid phantom. The phantom’s diameter and the height are 127 mm. Its thyroid simulator is a cylindrical hole on which a 15 ml vial is inserted. The acquisition and reconstruction parameters with the phantom were strictly the same as those used in clinical cases and described above. Only the vial contained radioactivity; the rest of the phantom was cold. The 20 mm depth at the location of the center of the vial corresponds to the thyroid depth in the neck of an average adult person (Fig. 1).

To ensure the linearity of the cross-calibration factor with the injected ^99m^Tc activity, various acquisitions were carried out in planar and tomographic modes, with radioactivity in the vial ranging from 1 to 20 MBq.

The cross-calibration factor *F*_cal_ in counts/(MBq.s) is given by:

$$F_{{{\text{cal}}}} = \frac{{N_{{{\text{ROI}}}} }}{{A_{{{\text{acq}}}} *\Delta t}}$$

where *N*_ROI_ is the number of counts in the region of interest and *A*_acq_ (MBq) is the mean activity of radioactivity decay in the vial over the acquisition duration *Δt* (s). Therefore, *A*_acq_ is given by the equation:

$$A_{{{\text{acq}}}} = \frac{{A_{0} .\mathop \int \nolimits_{0}^{\Delta t} e^{{ - \left( {{\mathbf{ln}}2} \right).{\varvec{t}}/{\varvec{T}}}} \cdot {\text{d}}t}}{\Delta t}$$

where *A*_0_ (MBq) is the initial activity, T(s) is the half-life of the ^99m^Tc. The quantification of activity was performed in the same way for the patients. More precisely, volumes of interest (VOIs) for Tomo-AC and Tomo-NoAC, and the regions of interest (2D ROIs) for Planar, were automatically segmented using a threshold of 40% of *P*_max_ [20] for SPECT and SPECT/CT VOIs, and 25% of *P*_max_ for Planar ROIs. *P*_max_ is defined as the maximum counts in a voxel (or pixel) within the region. In a preliminary study to validate these threshold values, a vial mimicking thyroid fixation (diameter 2.2 cm and height of 4 cm) was acquired with SPECT and planar acquisitions. The VOIs and 2D ROIs obtained of the vial images, with thresholds of 40% of *P*_max_ and 25% of *P*_max_, respectively, were in agreement with the vial’s geometric characteristics.

### Anthropomorphic phantom analysis

**Fig. 2:**
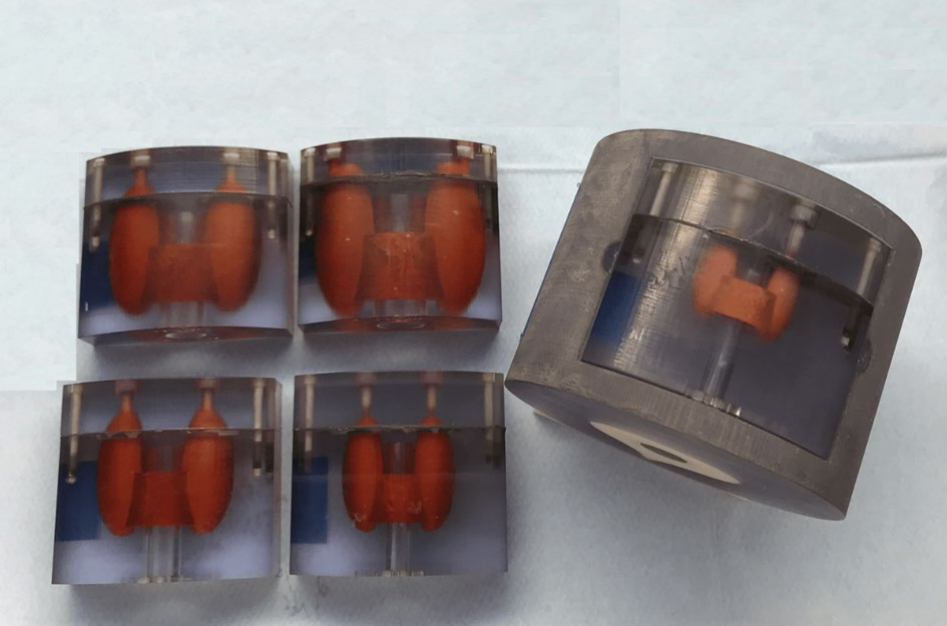
Five anthropomorphic thyroid phantoms made by IRSN (Institut de Radioprotection et de Sûreté Nucléaire). The phantom volume were 3.2, 7.5, 11.4, 19 and 30 cc and were introduced in the anthropomorphic neck phantom

Five anthropomorphic thyroid phantoms manufactured by IRSN (Institut de Radioprotection et de Sûreté Nucléaire) were imaged using the cross-calibration factor determined above to estimate the absolute thyroid activity (AThA). The thyroid volumes in the five phantoms were selected at 3.2, 7.5, 11.4, 19 and 30 cc, in alignment with ICRP recommendations for thyroid volumes and were inserted inside a neck phantom that contained a replica spinal cord and vertebral column (Fig. 2). These phantoms were designed with a realistic anthropomorphic shape. The phantoms were 3D printed using specific materials for an accurate simulation of the attenuation of biological tissues [21]. The tomography and planar acquisitions and reconstructions parameters were exactly the same as those used in clinical cases and described above. The range of activity in the ^99m^Tc solution injected into the thyroid phantoms was between 0.4 and 10 MBq. The theoretical absolute activity injected in the thyroid was defined as theoretical AThA. The estimation of the AThA was performed with the planar, Tomo-AC and Tomo-NoAC images.

The AThA was calculated as the following relation:

$${\text{AThA }}\left( {{\text{MBq}}} \right) = \frac{{N_{{\text{ROI,th}}} }}{{F_{{{\text{cal}}}} *\Delta t}}$$

where *F*_cal_ is the cross-calibration factor, *Δt* (s) the acquisition duration and *N*_ROI,th_ the counts in thyroid that were automatically segmented in the same way as the cross-calibration factor was determined.

### Measurement methodology of thyroid uptake

The thyroid uptake (TU) was calculated as the following relation:

$${\text{TU }}\left( {\text{\% }} \right) = \frac{{A_{{{\text{th}}}} }}{{A_{i} }}*100 = \frac{{N_{{\text{ROI,th}}} - N_{{{\text{back}}}} }}{{F_{{{\text{cal}}}} \cdot A_{i} }}*100$$

where *A*_th_ (MBq) is the activity measured in the thyroid ROI or VOI, *A*_*i*_ (MBq) is the activity injected to the patient and *F*_cal_ is the cross-calibration factor. The actual injected activity *A*_*i*_ was calculated by subtracting the activities of the full syringes with the empty ones.

The activity in the thyroid *A*_th_ was estimated in three different ways: static planar, SPECT and SPECT/CT acquisitions. The regions of interest, in which the counts in thyroid *N*_ROI,th_ were measured, were automatically segmented in the same way as for the determination of the cross-calibration factor: a threshold of 40% of *P*_max_ was chosen for the VOI on SPECT and SPECT/CT images, and a threshold of 25% of *P*_max_ was chosen for the 2D ROI on planar images. Additionally, a background region of interest (ROI_back_) was drawn underneath the thyroid in order to obtain a background corrected 2D ROI Thyroid counts for planar acquisitions. This background region of interest was not applied for the SPECT images.

Then for SPECT acquisitions: ${\text{TU }}\left( {\text{\% }} \right) = \frac{{N_{{\text{ROI,th}}} }}{{F_{{{\text{cal}}}} \cdot A_{i} }}*100$.

### Subjects’ inclusion and exclusion criteria

We compared the TU measurements on our CZT Gamma Camera with Planar, SPECT and SPECT/CT acquisition modes on a selected population of 23 patients. Twenty-three consecutive patients with hyperthyroidism, referred to our institution, were included in the study from October 2019 to June 2020. After the presumptive diagnosis was made, all subjects underwent a thyroid scintigraphy. Pregnancy was the exclusion criterion.

Our routine thyroid examination started with the planar acquisition 20 min after the injection and was followed immediately with a SPECT/CT acquisition. The ^99m^TcO_4_ uptake was measured 20 min after tracer intravenous injection. Residual activities in the syringes after injection were considered. The reference activity of the injected ^99m^TcO_4_ was 80 MBq.

Ethical approval was waived by the local Ethics Committee of our institution in view of the nature of the study and all the procedures being performed were part of the routine care. The retrospective study was registered with ClinicalTrials.gov number NCT05049551.

### Statistical analysis

Measured data were provided as the mean ± standard deviation (SD). Correlations of TU between Planar, Tomo-AC and Tomo-NoAC images and between theoretical and estimated AThA were assessed using the Pearson correlation coefficient (*r*). The difference was calculated between each of the TU measurements (Planar vs Tomo-AC, Planar vs Tomo-NoAC and Tomo-AC vs Tomo-NoAC) using a graphical Bland and Altman method. The graphical correlation analysis of the AThA was done individually on the five anthropomorphic 3D-thyroid phantoms. The relative percentage difference between theoretical and estimated AThA defined as 100*(AThA_Estimated_ − AThA_Theoretical_)/AThA_Theoretical_ for Planar, Tomo-AC and Tomo-NoAC were calculated for all the five anthropomorphic 3D-thyroid phantoms.

The upper and lower limits for the Bland and Altman plot analysis were calculated using mean ± 2 × SD. *P* value < 0.05 was considered statistically significant.

### Dosimetry estimation

For each patient, the dosimetric information included the volume computed tomography dose index for body in mGy (CTDI_vol_) and dose length product in mGy.cm (DLP) for the X-ray CT, and the activity injected of ^99m^TcO_4_ (in MBq) for the scintigraphy.

Effective dose (in Sv) and thyroid absorbed dose (in Gy) were calculated using International Commission on Radiological Protection (ICRP) reports 80 and 103 and the VirtualDose^TM^CT software from Virtual Phantoms, Inc [22].

## Results

### Cross-calibration parameters of the CZT gamma camera

**Fig. 3:**
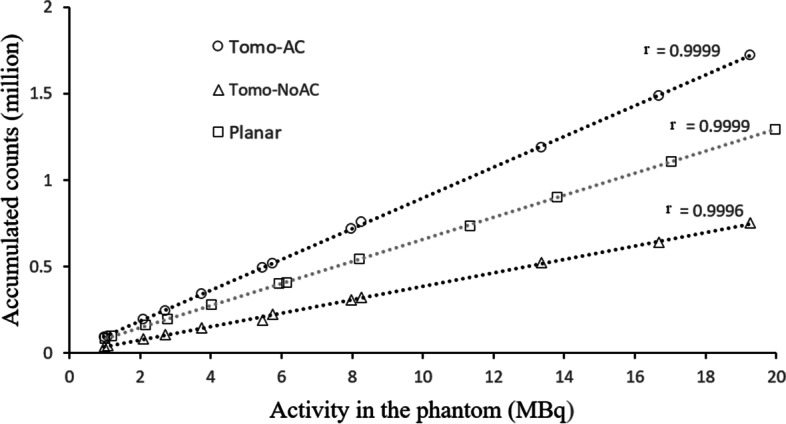
Representation of the variation of the accumulated counts as a function of the activity in the vial ranging from 1 to 20 MBq for the SPECT/CT (Tomo-AC), SPECT (Tomo-NoAC) and Planar acquisitions

The cross-calibration factors for the three acquisition modes: Planar, Tomo-AC and Tomo-NoAC, are summarized in Table 1. To ensure the stability of these cross-calibration factors, several activities were studied corresponding to a range of 1–20 MBq (Fig. 3). We verified the linear correlation coefficient (*r*) as 0.9999 for Planar, 0.9999 for Tomo-AC and 0.9996 for Tomo-NoAC, which correspond to a perfect linearity.

**Table 1:** Cross-calibration factors for the planar, SPECT/CT and SPECT acquisitions

Acquisition	Cross-calibration factors (counts.MBq^−1^ s^−1^)
Type	Mean ± SD
Planar	66.2 ± 4.9
SPECT/CT (Tomo-AC)	60.7 ± 0.7
SPECT (Tomo-NoAC)	26.5 ± 0.3

### Measurements of the absolute thyroid activity

**Fig. 4:**
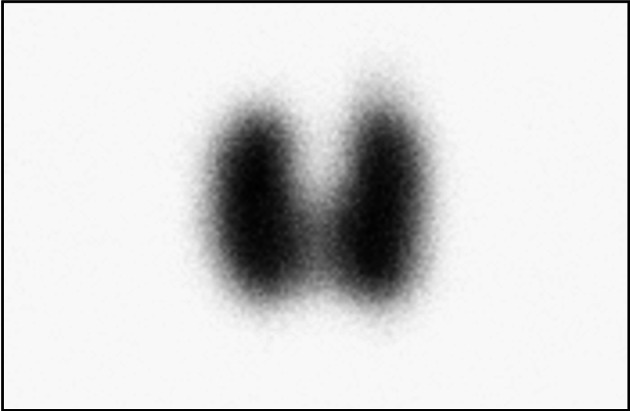
Planar scintigraphy of the 19 cc anthropomorphic thyroid phantom

**Fig. 5:**
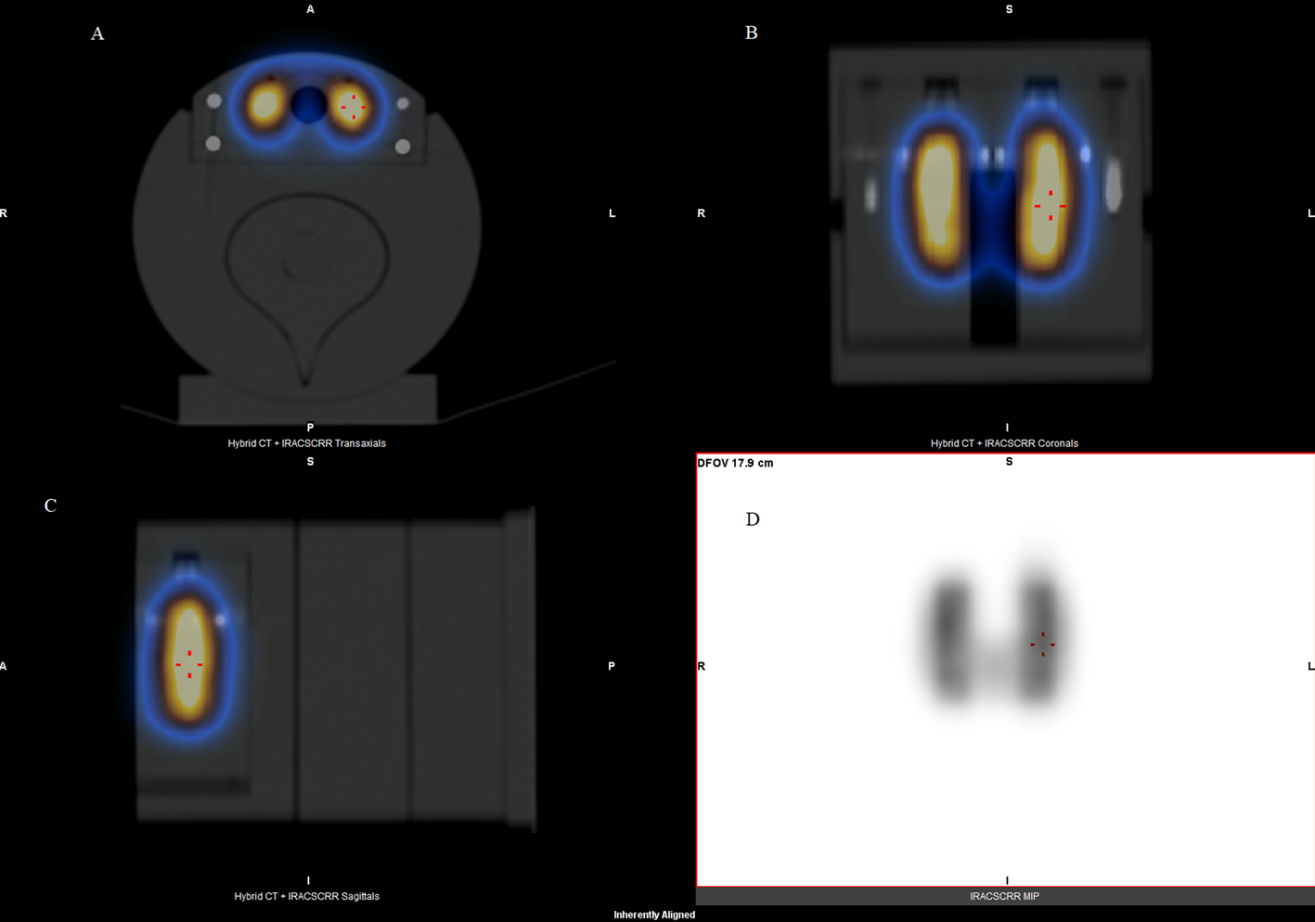
SPECT/CT (**A**: transverse slice, **B**: coronal slice, **C**: frontal slice) fusion views and maximum intensity projection view (**D**) of the 19 cc anthropomorphic thyroid phantom

**Fig. 6:**
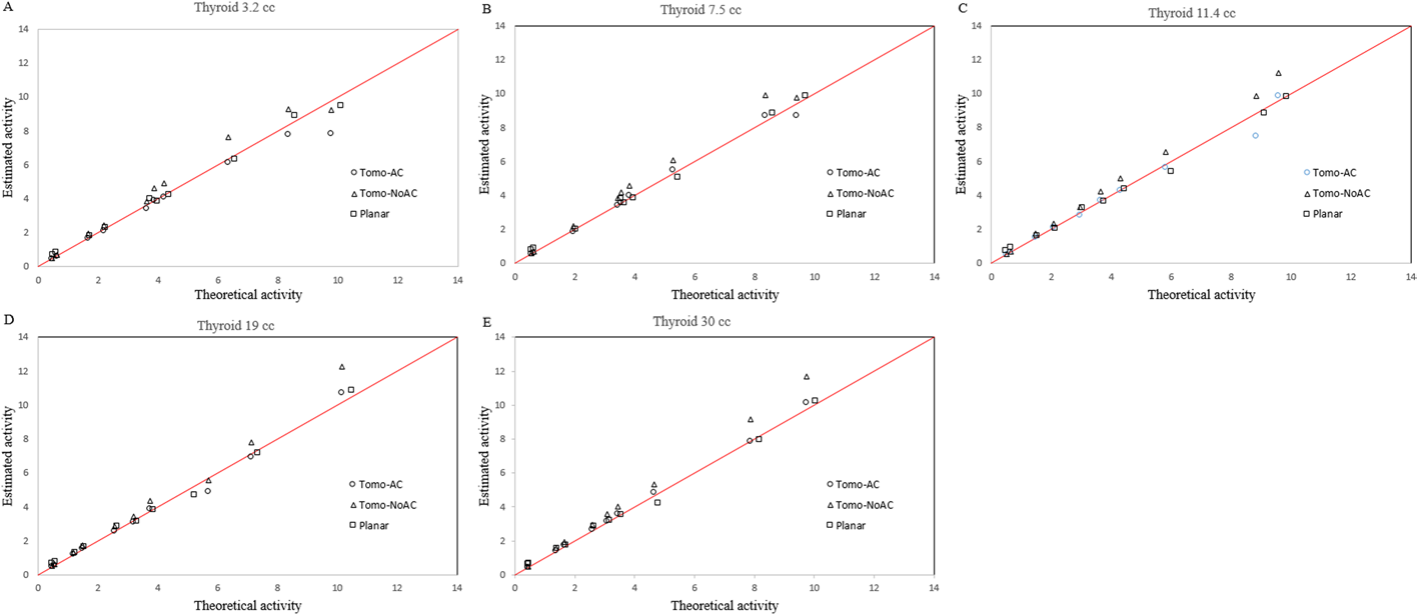
Correlations curves (**A**, **B**, **C**, **D** and **E**) between theoretical and estimated activity of Tomo-AC, Tomo-NoAC and Planar acquisitions for anthropomorphic thyroid volumes of 3.2, 7.5, 11.4, 19 and 30 cc

To generate a geometric model of thyroid that represents a human thyroid as closely as possible, Planar (Fig. 4) and SPECT/CT (Fig. 5) images of the anthropomorphic phantoms were acquired. For all five thyroids phantoms, the estimated AThA was plotted in comparison with the theoretical AThA for the three acquisition modes, as illustrated in Fig. 6. The injected activity in the five thyroid phantoms ranged from 0.4 to 10 MBq.

The Pearson correlation coefficient *r* for the theoretical and estimated AThA for Planar, Tomo-AC and Tomo-NoAC images were statistically highly correlated (*r* < 0.99; *P* < 10^–4^).

The average of the relative percentage difference between theoretical and estimated AThA for all the thyroid phantoms showed an excellent agreement and were for Planar, Tomo-AC and Tomo-NoAC images (8.6 ± 17.8), (− 1.3 ± 5.2) and (12.8 ± 5.7), respectively.

### Patients’ description

The twenty-three patients included 10 females (44%) and 13 males (56%). Their mean age was 58.9 ± 17 years, with a range of 29–89 years. Their mean weight was 73.1 ± 19.3 kg, with a range of 48–112 kg. The mean net injected activity of ^99m^TcO_4_ was 79.2 ± 3.7 MBq.

### Clinical results

**Fig. 7:**
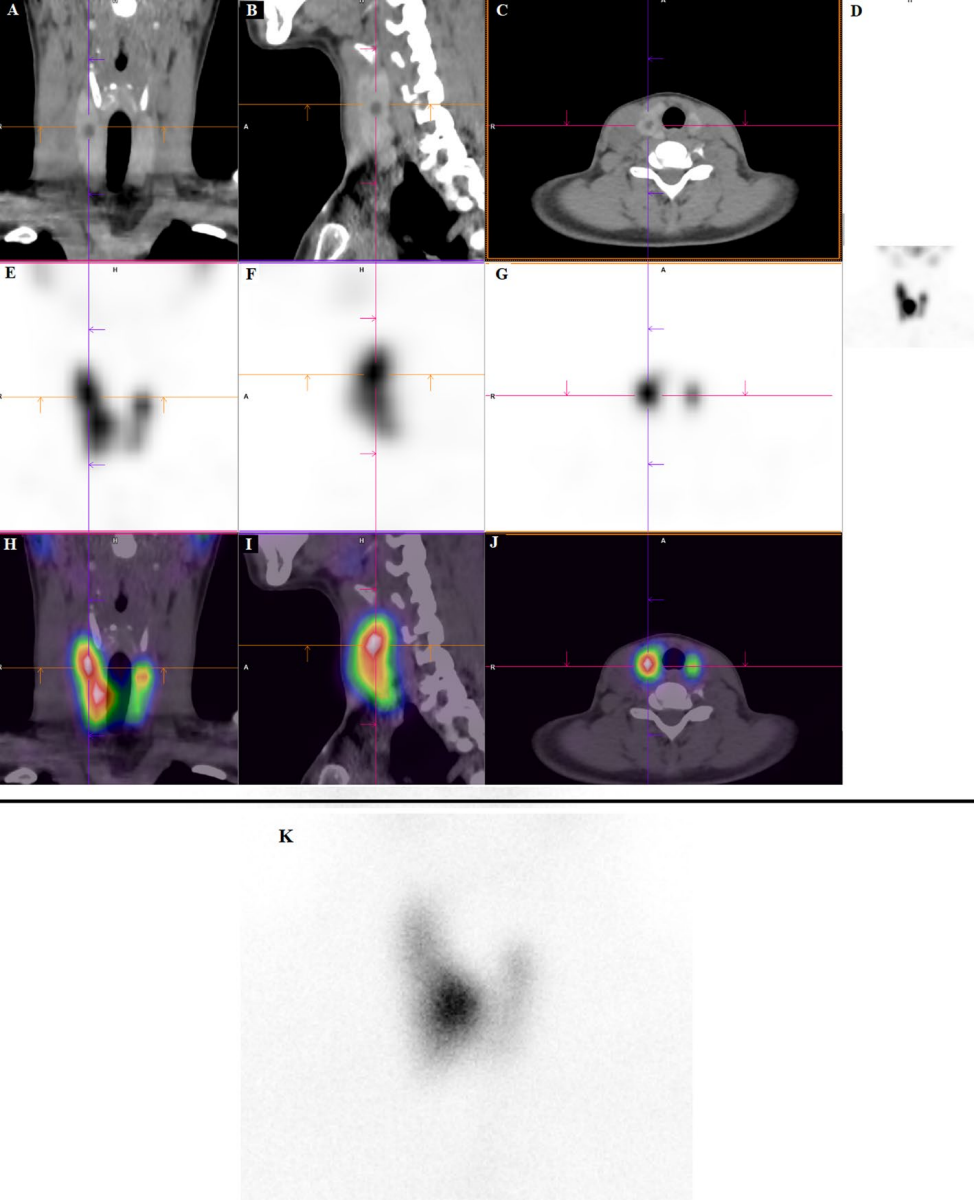
CT, SPECT and fusion in, respectively, coronal (**A**, **E**, **H**), sagittal (**B**, **F**, **I**), transvers (**C**, **G**, **J**) images, MIP (**D**) and Planar scintigraphy (**K**) of patient case #23, showing for Planar mainly the hot right nodule and for SPECT/CT the same hot right nodule and also hot nodules in the upper pole of right and left lobes

Thirteen patients (56%) were found to have one or several hot nodules. Among this subset of patients, SPECT/CT was able to detect all nodules seen with planar images. It was easier to localize nodules with a 3D representation of the thyroid in SPECT/CT images than planar image, as illustrated in Fig. 7 of the same patient (case #23). In Fig. 7K, the planar scintigraphy indicates a pathological, principally a large hot nodule in the right para-isthmic region. SPECT/CT, in contrast, reveals not only the large right para-isthmic hot nodule, but also two additional areas of focal uptake in the upper pole of both lobes, which are consistent with hot nodules (Fig. 7A, B, C, D, E, F, G, H, I, J).

### Measurements of the patients’ thyroid uptake

**Fig. 8:**
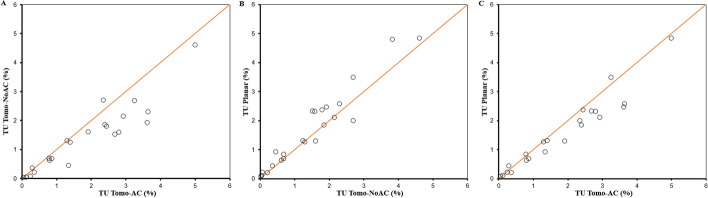
Correlations between TU based on different pairs of images Tomo-AC versus Tomo-NoAC (**A**), Planar versus Tomo-NoAC (**B**) and Planar versus Tomo-AC (**C**)

**Fig. 9:**
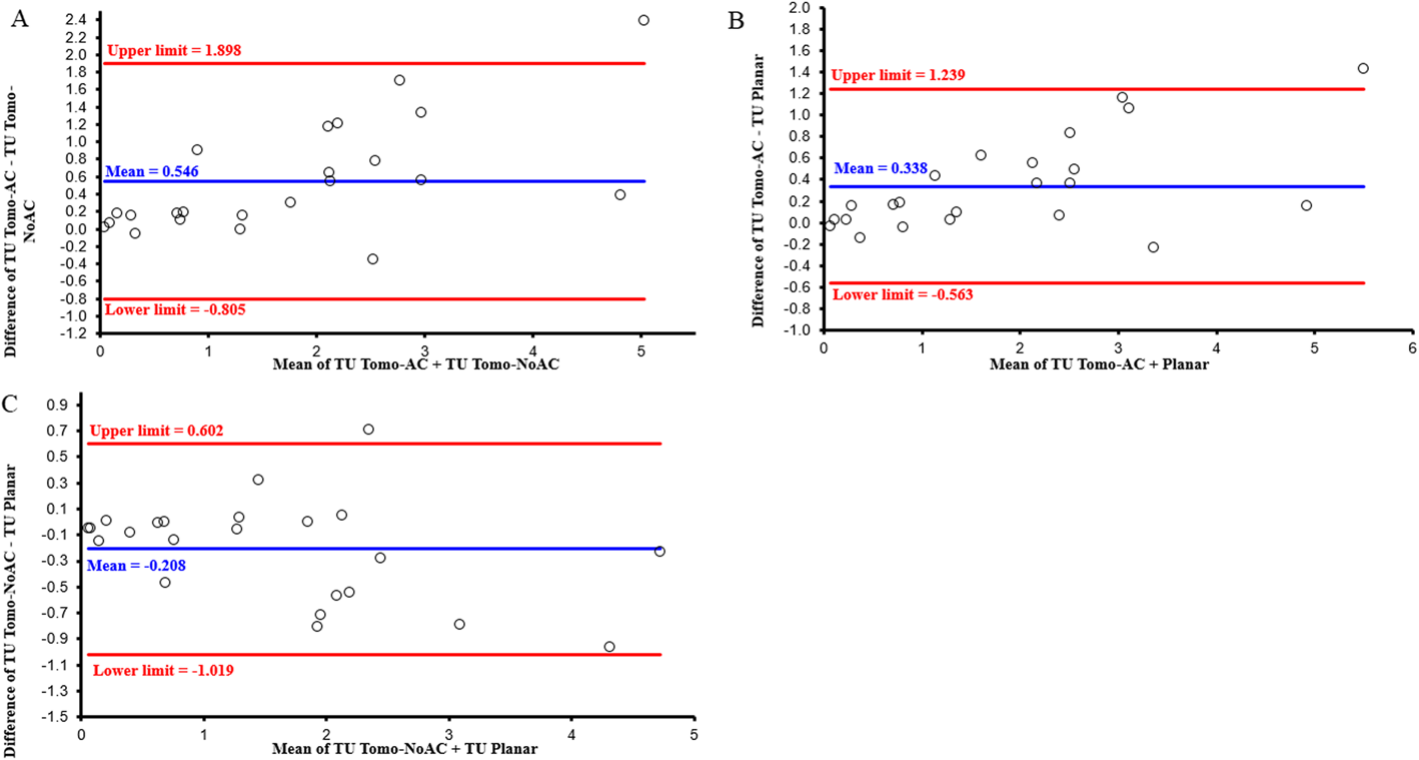
Bland and Altman plot analysis of TU calculated between Tomo-AC versus Tomo-NoAC (**A**), Planar versus Tomo-AC (**B**), and Planar versus Tomo-NoAC (**C**) images

Comparisons of TU between different pairs of images (Planar vs Tomo-AC, Planar vs Tomo-NoAC and Tomo-AC vs Tomo-NoAC) are shown in Fig. 8. Planar, SPECT/CT and SPECT images show statistically significant correlations (*r* = 0.972, 0.961 and 0.935, respectively; *P* < 10^–3^). The Bland and Altman analysis, as represented in Fig. 9, indicates a good agreement of the calculated TU between the Planar versus Tomo-AC, Planar versus Tomo-NoAC and Tomo-AC versus Tomo-NoAC images, with a mean difference, respectively, of 0.338 (− 0.563 to 1.239), − 0.208 (− 1.019 to 0.602) and 0.546 (− 0.805 to 1.898).

### Dosimetry results

Dosimetric information from CT and SPECT examinations of the 23 patients were used to calculate the thyroid absorbed dose. The mean activity of the injected ^99m^TcO_4_ was 79.2 ± 3.7 MBq. The mean of the CTDI_vol_ and DLP were estimated at 2.21 ± 0.2 mGy and 49.5 ± 9 mGy.cm, respectively. The mean effective dose and the thyroid absorbed dose, for the CT and the nuclear medicine (NM) parts, were found to be 0.34_CT_ + 0.95_NM_ mSv and 3.88_CT_ + 1.75_NM_ mGy, respectively. All of these results are summarized in Table 2.

**Table 2:** Thyroid and effective dosimetry data of the 23 patients

	Mean	SD	Range
Injected Activity (MBq)	79.2	3.7	(71.5–86.7)
CTDI_vol_ (mGy) (body)	2.21	0.2	(1.6–2.5)
DL P(mGy cm)	49.5	9	(33–65)
CT-effective dose (mSv)*	0.34	0.03	(0.24–0.38)
99mTcO_4_-effective dose(mSv)**	0.95	0.04	(0.86–1.04)
CT-Thyroid absorbed dose (mGy)*	3.88	0.36	(2.82–4.38)
99mTcO_4_-thyroid absorbed dose (mGy)**	1.74	0.08	(1.57–1.91)

*ICRP-103 and Virtual-Phantoms, Inc. software, ** ICRP-80

## Discussion

In order to quantify ^99m^TcO_4_ TU on planar, SPECT and SPECT/CT series using a large field of view CZT gamma camera, we first determined cross-calibration factors using a thyroid phantom. Then, the AThA was calculated for planar and SPECT/CT acquisitions on five anthropomorphic 3D thyroid phantoms, and the TU was calculated for the acquisitions performed on 23 consecutive patients. To our knowledge, the feasibility of such thyroid quantification using a large field of view CZT gamma camera has not been previously demonstrated in the literature.

The TU value obtained with planar acquisition after injection of ^99m^TcO_4_ is one of the parameters used in nuclear medicine for the diagnosis of thyroid diseases and for pathological follow-up. Its clinical value is limited, as it depends on many factors such as thyroid volume, patient’s iodine supply, hormonal status and patient’s age [3–5, 18, 23, 24]. Its use is recommended by the European Association of Nuclear Medicine (EANM) and the Society of Nuclear Medicine and Molecular Imaging (SNMMI) practice guideline for thyroid scintigraphy [18].

However, one of the main issues with this parameter is that its normal reference range is not clearly defined [25–30]. Estimated of TU and thyroid scintigraphy are established according to guidelines [18, 19] using planar acquisitions on a NaI scintillation detector equipped with an Anger-type gamma camera with parallel and pinhole collimators. The pinhole collimator is used just for diagnostics, but not for the estimation of TU. Attenuation correction (AC) and scatter correction (SC), resolution recovery (RR) and the iterative OSEM algorithm allow a quantitative SPECT/CT approach [12, 31–33], despite the relatively poor spatial resolution and the partial volume effects that limit quantitation of radioactivity for objects smaller than three times the spatial resolution [34].

Ahmed et al. performed a bibliographic evaluation [35] with iodine-131 on hybrid SPECT/CT imaging compared to planar scintigraphy. The aim of that study was to treat differentiated thyroid carcinomas. The authors concluded that SPECT/CT was superior to planar in distinguishing pathologies from physiological uptake by increasing the accuracy of image interpretation. SPECT/CT improved the accuracy of differentiating the stages of thyroid cancer and subsequent patient management. Their conclusion was mainly based on the lack of anatomical detail in planar gamma cameras images and the superimposition of areas leading to false positive results and potential to over-treat patients.

Zaidi [36] investigated comparative methods on a thyroid phantom to quantify thyroid volume using planar and SPECT imaging with technetium-99 m on a scintillation (NaI) gamma camera. He concluded that images from SPECT with attenuation and scatter corrections provided the most accurate determination of thyroid phantom volume, but failed at smaller volumes where errors caused by the partial volume effect were significant.

In contrast, Iizuka et al. [37], using iodine-131 scintigraphy, compared planar with SPECT images for quantification of radiation intensity, concluding that planar images provided better accuracy for determining the radiation dose. Their approach differed from ours and was based on an iodine-131 reference capsule placed next to the patient during image acquisition. Iodine-131, as a high photon energy radioisotope, has poor sensitivity to the gamma camera due to collimator penetration, a factor that could explain those results.

The CZT digital detector, based on cadmium zinc telluride technology, provides high resolution SPECT images by directly converting gamma radiation into an electrical signal. The high performance of the CZT detector with its specific acquisition and reconstruction parameters (WEHR collimator, energy window set at 15%, iterative reconstruction with AC, SC and RR corrections) provides several major advantages for our purpose. First, it was not necessary to increase the injected ^99m^TcO_4_ activity while maintaining the quantitative accuracy of the SPECT and SPECT/CT image data [32]. Second, the high spatial and energy resolutions of the CZT, compared to the Anger camera, result in an improved image quality in terms of contrast and spatial resolution [17].

The perfect linearity of the change in accumulated counts as a function of activity for Planar, Tomo-AC and Tomo-NoAC images, with *r* = 0.9999, 0.9999 and 0.9996, respectively (Fig. 3), ensures the stability of the cross-calibration factors. Estimation of the AThA of the five anthropomorphic phantoms show evidence that the cross-calibration factors are validated for all five thyroid sizes (from 3.2 cc to 30 cc) and for variable activity in the phantoms ranging from 0.4 to 10 MBq, which represents the typical uptake of ^99m^TcO_4_ activity in a patient thyroid.

The average of the relative percentage difference between theoretical and estimated AThA for all the thyroid phantoms showed an excellent agreement, particularly for the Tomo-AC, and were for Planar, Tomo-AC and Tomo-NoAC images (8.6 ± 17.8), (− 1.3 ± 5.2) and (12.8 ± 5.7), respectively. The results in Figs. 8 and 9 show a good agreement among the TU values calculated with planar and tomographic images using cross-calibration factors. In addition, the correlation between Planar versus Tomo-AC, Planar versus Tomo-NoAC and Tomo-AC versus Tomo-NoAC (Fig. 8) was excellent (*r* = 0.972, 0.961 and 0.935, respectively; *P* < 10^–3^), offering the possibility of using either SPECT or SPECT/CT for TU quantifications. The thyroid’s proximity to the skin, inducing a low level of attenuation and scattering, partly explains the excellent correlation values between Planar, SPECT and SPECT/CT images. In addition, SPECT imaged limited patient exposure compared to SPECT/CT.

Regarding the contribution of 3D imaging (Tomo-AC and NoAC), although it did not directly influence the therapeutic strategy in our 23 cases, a potential superiority of tomographic images in the detectability and localization of thyroid nodules in comparison with planar conventional acquisitions was evident. This finding is consistent with previous studies [12, 20, 31, 32, 35, 36, 38] that have demonstrated the utility of SPECT for accurate localization of thyroid tracer uptake. Clearly, the CT scan associated with SPECT enhances the anatomic localization of hot thyroid nodules and could likely be relevant to patient care.

A factor of consideration in this study’s procedure is the potential radiation exposure to patients with CT acquisitions, especially for the thyroid glands. Nonetheless, in our configuration of a CZT coupled with an efficient CT scan, we were able to inject a low activity of ^99m^TcO_4_ around 79.2 ± 3.7 MBq. Using low activity was possible due to the high performance of the CZT technology, along with specific SPECT reconstruction parameters and corrections. In addition, we used a low tube voltage (100 kVp) and the CT reducing methods such as current modulation (smart mA) and iterative CT reconstruction (ASIR).

The estimated effective dose of the patients was 1.29 mSv: 0.34 mSv for CT plus 0.95 mSv for the injection of ^99m^TcO_4_. This is an acceptable effective dose, considering that the annual individual exposure among the population in France is 4.5 mSv [39] and the typical effective dose of an abdomen CT is 8.1 mSv [40]. However, the total organ absorbed dose for the thyroid was 5.63 mGy: 3.88 mGy for CT plus 1.74 mGy for the injection of ^99m^TcO_4_ which should definitely motivate to reduce radiation exposure, especially for CT.

## Conclusion

Using CZT technology, cross-calibration factors, validated through anthropomorphic thyroid phantoms using the same acquisition conditions as the patients, provide the ability to validate the TU value on a small series of patients with SPECT and SPECT/CT images. The Planar gold standard acquisition for TU could be substituted by SPECT or SPECT/CT acquisitions.

Although CT for AC reduces the errors on estimated AThA as compared to no AC and CT combined with SPECT enhances the anatomic localization of hot thyroid nodules, the additional value of combined CT remains to be confirmed in larger studies and involves an increase in the exposure. Patient radiation exposure remains a concern, especially because the thyroid is considered as a highly sensitive organ to ionizing radiation.

## Data Availability

The datasets used and analyzed during this current study are available from the corresponding author upon reasonable request.

## Abbreviations

AC: Attenuation correction
*A*_i_: Activity injected to the patient
ANSI: American National Standards Institute
ASIR: Adaptive statistical iterative reconstruction
*A*_th_: Activity measured in the thyroid
AThA: Absolute thyroid activity
CT: X-ray commuted tomography
CTDI: Computed tomography dose index
CZT: Cadmium Zinc Telluride
DLP: Dose length product
EANM: European Association of Nuclear Medicine
*F*_cal_: Cross-calibration factor
Gy: Gray
IAEA: International Atomic Energy Agency
ICRP: International Commission on Radiological Protection
MIP: Maximum Intensity Projection
N_ROI,th_: Regions of interest counts in thyroid
OSEM: Ordered subsets expectation minimization
PET: Positron emission tomography
*P*_max_: Maximum counts in a voxel (or pixel) within the region
*r*: Pearson correlation coefficient
ROIs: Regions of interest
ROI_back_: Background region of interest
RR: Recovery reconstruction
SC: Scatter correction
SD: Standard deviation
SNMMI: Society of Nuclear Medicine and Molecular Imaging
SPECT: Single photon emission computed tomography
Sv: Sievert
Tomo-AC: Tomography with attenuation correction
Tomo-NoAC: Tomography without attenuation correction
TU: Thyroid uptake
VOIs: Volumes of interest
VS: Versus
WEHR: Wide Energy High Resolution
